# P-1858. Real-world Experience of Rezafungin in an Outpatient Infusion Center Upon Discharge

**DOI:** 10.1093/ofid/ofaf695.2027

**Published:** 2026-01-11

**Authors:** Jill Foster, Christopher M Bland, Bruce M Jones

**Affiliations:** University of Georgia, Savannah, GA; University of Georgia College of Pharmacy, Savannah, GA; St. Joseph's/Candler Health System, Savannah, GA

## Abstract

**Background:**

Rezafungin is a long-acting echinocandin with a half-life > 130 hours, allowing once-weekly dosing that has the potential for financial and logistical advantages. It is indicated for adults for the treatment of candidemia/invasive candidiasis. Many patients are not candidates for azole therapy due to drug interactions or resistance leaving daily intravenous echinocandins for treatment. This study assessed rezafungin use in patients who were discharged from a community health system to receive weekly treatment at an outpatient infusion center.

**Table 1:**
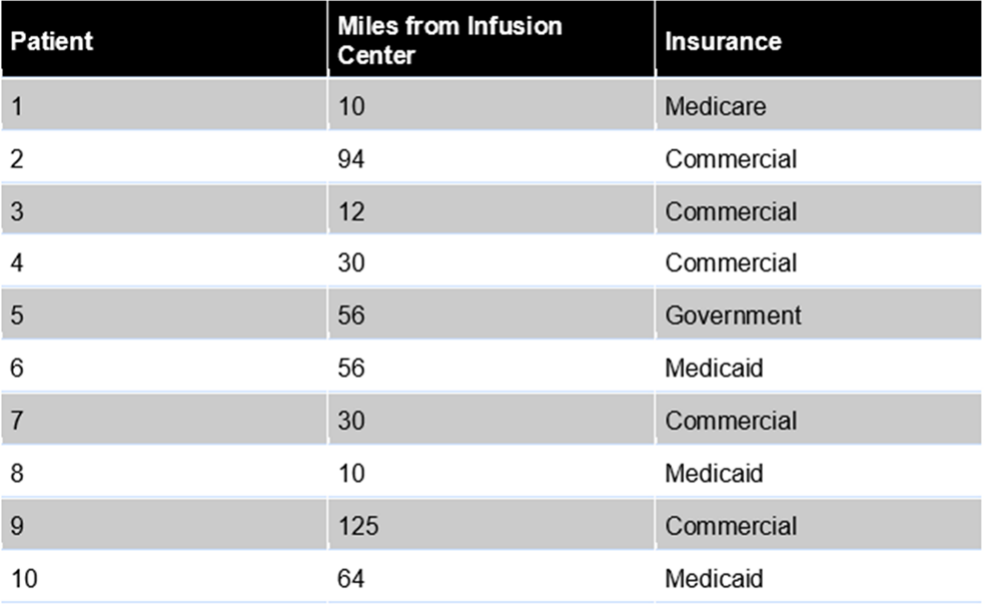
Patient Residence and Insurance Demographics

**Table 2:**
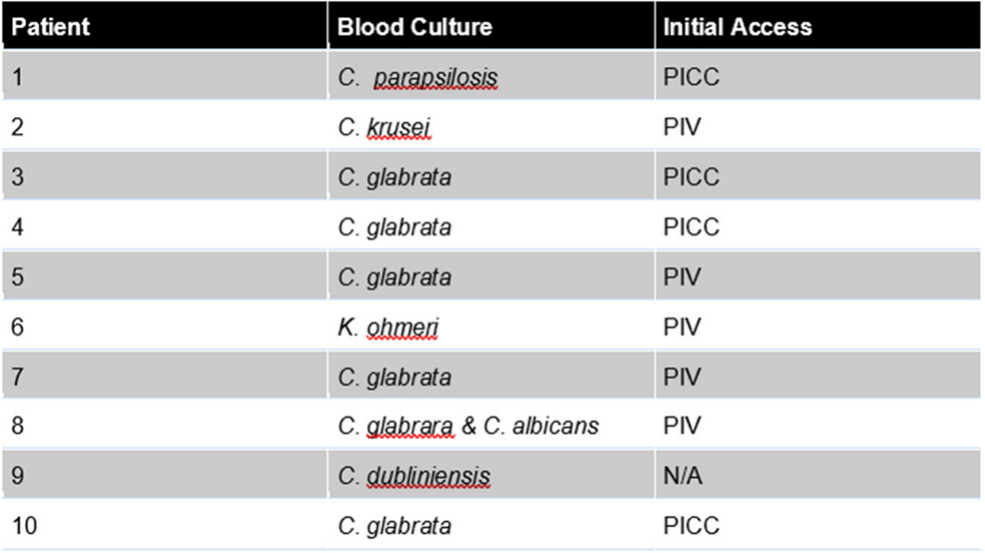
Microbiology and IV Access Type

**Methods:**

This was a retrospective chart review of adult patients who received ≥1 outpatient infusion(s) of rezafungin after discharge from January 2024 - February 2025. Baseline demographics, infectious diagnosis and previous treatment, microbiology, adverse events, hospital readmissions, and relevant financial data was collected. Data was recorded and compared in Microsoft Excel using descriptive statistics. The primary objective was to evaluate 30 day readmission after infusion, patient characteristics and overall financial impact to the patient and health system.

**Results:**

Ten patients received rezafungin in the outpatient setting after discharge and were included. Two patients had a 30 day readmission with non-fungal related conditions. Mean age was 54 years (36-71) with 6 being female. Patients lived a median of 43 miles from the infusion center and were split evenly between public and private insurance (Table 1). Most patients grew Candida spp., with initial organism growth and access reported in Table 2. Initial treatment regimens included micafungin (6) and fluconazole (2). Median duration of therapy (DOT) prior to rezafungin was 8 days (4-20) of micafungin and 2 days (1-3) of fluconazole. One patient discontinued fluconazole due to prolonged QTc interval. Median DOT for rezafungin was 14 days (7-63), with an average 2 doses received. One infusion-related adverse reaction of body numbness and shortness of breath was reported, but was resolved by slowing the infusion rate. Insurance reimbursement data is currently being collected for assessment.

**Conclusion:**

Discharging patients to receive rezafungin as an outpatient infusion was overall well tolerated and logistically may offer an advantage to patients and institutions.

**Disclosures:**

Christopher M. Bland, PharmD, FCCP, FIDSA, BCPS, Merck: Honoraria|Nestle Health Sciences: Honoraria|Shionogi, Inc.: Advisor/Consultant|Shionogi, Inc.: Honoraria Bruce M. Jones, Pharm.D., FIDSA, BCPS, AbbVie: Advisor/Consultant|AbbVie: Honoraria|Ferring: Grant/Research Support|Ferring: Honoraria|Innoviva: Honoraria|Paratek: Advisor/Consultant|Paratek: Honoraria

